# Michigan Monthly Bulletin of Vital Statistics

**Published:** 1898-11

**Authors:** 


### BOOK NOTICES.

Michigan Monthly Bulletin of Vital Statistics. Published by the Department of State, Lansing, Michigan. Washington Gardner, Secretary of State; Samuel A. Kennedy, Deputy Secretary. Edited by Cressy L. Wilbur, M. D., Chief of Division of Vital Statistics.

The thirteenth issue of this publication urges the use of a plan for uniform statistics of mortality for the whole world. It speaks of the action of the American Public Health Association in adopting a uniform system of classification of the causes of death for the three countries represented in this association, namely, United States, Canada, and Mexico, and urges its adoption for the whole world. The system advocated is the Bertillon system. What this system is can be found by perusing this and the previous numbers of the *Bulletin*. The other pages are devoted to the vital statistics of the State of Michigan.

